# A comparison of clinical pathologic characteristics between alpha-fetoprotein negative and positive hepatocellular carcinoma patients from Eastern and Southern China

**DOI:** 10.1186/s12876-022-02279-w

**Published:** 2022-04-23

**Authors:** Xiaowei Chi, Liejun Jiang, Yulin Yuan, Xinyan Huang, Xuemei Yang, Steven Hochwald, Jie Liu, Huayi Huang

**Affiliations:** 1 Department of Laboratory Medicine, The Affiliated Hospital of Shandong University of Traditional Chinese Medicine, 42 Wenhua West Rd, Jinan, 250012 Shandong China; 2 Department of Laboratory Medicine, The People’s Hospital of Guangxi Zhuang Autonomous Region, 6 Taoyuan Rd, Nanning, 530021 Guangxi China; 3 Department of Nuclear Medicine, The People’s Hospital of Guangxi Zhuang Autonomous Region, 6 Taoyuan Rd, Nanning, 530021 Guangxi China; 4 Department of Surgical Oncology, Roswell Park Comprehensive Cancer Center, Elm and Carton Streets, Buffalo, NY 14263 USA; 5 Department of Laboratory Medicine, Jinan Central Hospital, 105 Jiefang Rd, Jinan, 250013 Shandong China; 6 School of Medical Laboratory, Youjiang Medical University for Nationalities, No. 98 Chengxiang Road, Baise, 533000 Guangxi China; 7 Mindray North America, 800 MacArthur Boulevard, Mahwah, NJ 07430 USA

**Keywords:** Alpha-fetoprotein (AFP), Hepatocellular carcinoma (HCC), Liver cirrhosis, Chronic hepatitis B, Protein induced vitamin K absence or antagonist-II (PIVKA-II), Hepatitis B virus DNA (HBV DNA), Carcinoembryonic antigen (CEA), Neutrophil to lymphocyte ratio (NLR)

## Abstract

**Background:**

Alpha-fetoprotein (AFP) is a biomarker used in clinical management of hepatocellular carcinoma (HCC), however, approximately 40% of HCC patients do not present with elevated serum AFP levels. This study aimed to investigate the clinical and pathologic characteristics between AFP positive and negative HCC patients to allow for improved clinical management and prognostication of the disease.

**Methods:**

This study observed a cohort of HCC patients from Eastern and Southern China with comparisons of the clinical and pathologic features between serum AFP positive and negative patient groups; patients with decompensated hepatic cirrhosis, those with chronic hepatitis B, and hepatitis B virus (HBV) asymptomatic carrier patients were used as controls. Data included the laboratory results, pathology diagnosis, clinical staging and scores were obtained from routine clinical diagnostic methods.

**Results:**

Patients with HCC, larger tumor sizes, liver cancer with hepatic cirrhosis, portal vein thrombosis, metastasis, high Child–Pugh score, high Barcelona-Clínic Liver Cancer (BCLC) stage, and advanced clinical stage had significantly higher serum AFP levels. Also, patients with HBsAg and HBeAg positive, high HBV DNA levels had significantly higher serum AFP levels. Patients with high serum AFP levels had higher protein induced by vitamin K absence or antagonist-II (PIVKA-II), alanine aminotransferase (ALT), aspartate aminotransferase (AST), alpha-l-fucosidase (AFU), gamma-glutamyl transpeptidase (γ-GT), γ-GT /ALT, direct bilirubin (DBIL), indirect bilirubin (IDBIL), fibrinogen, and D-dimer levels. Patients with AFP positive had higher white blood cells (WBC), neutrophil, monocyte, and platelet count and neutrophil to lymphocyte ratio (NLR).

**Conclusions:**

The are significant differences in clinical pathologic characteristics between AFP positive and negative HCC patients which may be helpful for the management and prognostication of the disease.

**Supplementary Information:**

The online version contains supplementary material available at 10.1186/s12876-022-02279-w.

## Introduction

Hepatocellular carcinoma (HCC) is a common malignancy in Asia [1]. About 364,800 new cases of liver cancer were diagnosed in China, resulting in about 318,800 deaths in 2014 [2]. The incidence of liver cancer in China is mainly related to hepatitis B virus infection, hepatic fibrosis, and biliary cirrhosis. Treatments for liver cancer include surgical resection, interventional embolization, radiofrequency ablation, liver transplantation, and chemotherapy. However, the postoperative recurrence and metastases rates remain high and the 5 years survival rate is still very low [3, 4]. The bottle neck for long-term survival improvement is the inability for the diagnosis of early stages of the disease when the tumor size is under 3 cm. HCC screening in high-risk populations relies on serum tumor markers and ultrasonography. For serum biomarkers, the alpha-fetoprotein (AFP) is commonly used as a first line screening tool recommended by guidelines worldwide and is in clinical practice because of easy accessibility, non-invasive nature, and low cost. Serum AFP concentrations > 400 ng/ml is considered as reliable for supporting the diagnosis of hepatocellular carcinoma. However, about 30–40% of HCC are AFP-negative (< 20 ng/mL) [5, 6]. The mechanism of AFP repression is unknown, it is hypothesized that silencing of *AFP* promotors because of mutation or promoter inhibition is one of the reasons [7–9]. HCC patients with AFP negative or weak positive serum tests are usually consistent with the characteristics of high differentiated cancers. Although a negative serum AFP may be associated with favorable liver cancer outcomes, it results in a miss diagnosis of liver cancer in general [10, 11]. Efforts focusing on identifying new HCC serum biomarkers for risk evaluation, early diagnosis, recurrence, and prognosis prediction have been extensively studied, however, no new biomarkers are satisfactory thus far [12–16]. A study found that high neutrophil–lymphocyte ratio (NLR) was associated with low AFP expression in patients with hepatitis B virus (HBV) related HCC, therefore, NLR could be a potential clinical marker of the pathogenesis of AFP negative liver cancer [17]. Another study found that fibrinogen to pre-albumin ratio was gradually increased alongside with the development of AFP-negative HCC and positively correlated with tumor size and Barcelona Clinic Liver Cancer stages (BCLC) [18]. Thus, we hypothesize that serum biomarkers would vary between AFP-positive and negative HCC patients and could be indicators of liver cancer pathogenesis and possibly useful in clinical management and prognostication. In this study, we analyzed the laboratory parameters and clinical characteristics between AFP-positive and negative HCC patients from Eastern and Southern China to better understand the pathogenesis of the disease, improving the management, and prognostication.

## Patients and methods

### Patients

This study analyzed a total of 992 HCC patients from both Eastern and Southern China (861 males, 131 females, ages from 23 to 89 years) from 2016 to 2020. The diagnosis of HCC followed the 2019 Chinese clinical guidelines for the management of hepatocellular carcinoma: updates and insights [19, 20]. Since hepatitis B virus (HBV) infection is the major cause of HCC in China, this study detected the HBV DNA and it associated biomarkers in the HCC patients. Furthermore, the pathogenesis of HCC caused by different etiologies usually undergoes from chronic inflammation to liver cirrhosis, and then HCC. Thus, we included the liver cirrhosis patients as one of the control groups as well. All HCC patients met pretreatment requirement, patients transferred from other hospitals were also excluded. In addition, 121 patients with decompensated hepatic cirrhosis (DHC, 85 males, 36 females, ages from 30 to 86 years and 40 to 85 years, respectively), 114 with chronic hepatitis B (CHB, 80 males, 34 females, ages from 14 to 72 years and 21 to 68 years, respectively), and 127 with hepatitis B virus asymptomatic carriers (AsC, 68 males, 59 females, ages from 5 to 67 years and 25 to 80 years, respectively) were used as control groups. Diagnosis of DHC followed the EASL Clinical Practice Guidelines for the management of patients with decompensated cirrhosis [21], and diagnosis of CHB virus infection followed the EASL Clinical Practice Guidelines: Management of chronic hepatitis B virus infection and AASLD 2018 hepatitis B guidance [22, 23]. This study was approved by the Institute Review Board/Ethics Committee of The Affiliated Hospital of Shandong University of Traditional Chinese Medicine and The People’s Hospital of Guangxi Zhuang Autonomous Region for the collection of patients’ information, study protocols used, and clinical practice guidelines to be followed. Patients’ demographics are listed in Additional file 2: Table S1.

### Chemiluminescence immunoassay of serum biomarkers

Serum AFP, carcinoembryonic antigen (CEA), cancer antigen 125 (CA-125), and cancer antigen 199 (CA-199) were analyzed on the Cobas e801 platform (Roche, Rotkreuz, Switzerland) and UniCel DxI 800 Access (Beckman Coulter, Brea, California, USA); Protein induced by vitamin K absence or antagonist-II (PIVKA-II) was analyzed on the ARCHITECT i2000SR platform (Abbott Park, Illinois, USA); HBsAg and HBeAg were analyzed on the EasyCuta platform (PerkinElmer, Taicang, Suzhou, China) following the manufacturers’ instruction and laboratory standard operating procedures. Serum AFP level lower than 20 ng/mL was defined as AFP negative, while it was defined as AFP positive if the concentration was higher than 20 ng/mL.

### Clinical chemistry analysis

Serum homocysteine (Hcy), ferritin (SF), alanine transaminase (ALT), γ-glutamyl transpeptidase (γ-GT), aspartate aminotransferase (AST), alpha-L-fucosidase (AFU), direct bilirubin (DBIL), indirect bilirubin (IBIL), total protein (TP), and albumin (ALB) were analyzed on the AU5800 platform (Beckman Coulter, Brea, California, USA) following the manufacturer’s instruction and laboratory standard operating procedures.

### Hematology analysis

Hematology analysis of blood cells (complete blood count and differentiation) were performed on the XN 9000 hematology analyzer (Sysmex, Kobe, Japan) and BC 6900 hematology analyzer (Mindray, Shenzhen, China) following the manufacturers’ instruction and laboratory standard operating procedures.

### Coagulation analysis

Prothrombin time (PT), fibrinogen, and D-dimer were analyzed using the STA-R Evolution (Diagnostica Stago S.A.S., Asnières sur Seine Cedex, France) following the manufacturer’s instruction and laboratory standard operating procedures. The international normalized ratio (INR) was automatically calculated by the analyzer.

### Hepatitis B virus DNA detection

Hepatitis B virus (HBV) DNA was detected on the C1000 Touch Thermocycler (Bio-Rad, Hercules, California, USA) by using the real time polymerase chain reaction (PCR) technique following the manufacturer’s instruction and laboratory standard operating procedures. The cut-off value for DNA levels for analysis was set at 500 IU/mL.

### Pathology diagnosis

The tumor histologic types and differentiation were characterized by pathology examination of routine pathologic diagnosis from surgical tissues.

### Child–Pugh scoring and BCLC staging classification of hepatocellular carcinoma

The Child–Pugh Scoring was performed according to the scoring system [24]. The BCLC staging classification of hepatocellular carcinoma was used in routine clinical management [25].

### Statistical analysis

All data was analyzed using the SPSS version 26 and GraphPad Prism version 9 software. Since the data sets were skewed, thus, nonparametric tests (Mann–Whitney U Test, Kruskai–Wallis H Test, Spearman Test, and Pairwise comparison) were used for the analysis. A p value < 0.05 was considered significant.

## Results

### Normality test result of data set

The statistical analysis indicated that the data was skewed. The distribution of serum AFP in patient groups is shown in Additional file 1: Figure S1. Most patients’ AFP levels were at the lower end (left in the histogram of Additional file 1: Fig. S1).

### Comparison of serum AFP levels among patient groups

Table 1 lists the comparison of serum AFP levels among patient groups using the nonparametric method. Statistical results of pairwise comparison are shown in Additional file 1: Figure S2 A. The results show that serum AFP levels in HCC patients are significantly higher than in CHB, DHC, and AsC patients (p < 0.0001 for all). AFP level in CHB patients is significantly higher than in AsC patients (p < 0.0001); AFP level in DHC patients is significantly higher than in Asc patients (p = 0.0238); there is no significant difference in AFP level between CHB and DHC patients (p = 0.5050).

**Table 1:** Comparison of serum AFP levels among patient groups

	n	Median	P25–P75	Mean rank	Test statistic	*P*
HCC	992	177.80	9.25–1210.00	801.61	405.518	0.000
CHB	114	5.72	2.80–21.99	447.83		
DHC	121	4.41	2.16–11.53	359.79		
AsC	127	2.61	1.91–3.46	216.89		

HCC: Hepatocellular carcinoma; DHC: Decompensated hepatic cirrhosis; CHB: Chronic hepatitis B; AsC: Hepatitis B virus asymptomatic carrier

### Association between serum AFP levels and clinical pathologic features in HCC patients

Serum AFP levels in patients with tumor size ≥ 10 cm were significantly higher than that of < 10 cm (p = 0.000); there is no significant difference in AFP level between HCC patients with and without liver cirrhosis (p = 0.398). Patients with portal vein thrombosis had higher AFP levels (p = 0.000); patients who had metastasis also had higher AFP levels (p = 0.004); AFP levels in patients with large tumors were significantly higher than those with diffuse or single nodule tumors (p = 0.000); high Child–Pugh score patients had higher AFP levels than those with low scores (p = 0.000); patients with high BCLC stage had higher AFP levels than those with low stages (p = 0.000); similarly, patients with more advanced clinical stages had higher AFP levels than that of lower stages (p = 0.000). There was no significant correlation between tumor differentiation and serum AFP levels (p = 0.092) (Table 2). Additional file 1: Figure S2 B–E further show the pairwise comparison.

**Table 2:** Association between serum AFP levels and clinical pathologic features

		n	Median	P25–P75	Mean rank	Test statistic	*P*
Tumor size^a^	< 10 cm	569	82.53	7.08–1000	379.83	− 7.593	0.000
(largest in diameter)	≥ 10 cm	279	928.70	51.83–3000	515.59		
Liver cirrhosis^a^	No	439	211	7.31–1494.20	505.12	− 0.845	0.398
	Yes	553	160.7	11.29–1210.00	489.66		
PVT^a^	No	711	97.63	6.98–1000.00	459.53	− 6.472	0.000
	Yes	281	844.1	49.49–1914.15	590.04		
Metastasis^a^	No	699	138.00	7.60–1210	479.39	− 2.909	0.004
	Yes	293	390.10	17.67–1210	537.33		
Types of tumor^b^	Single nodule	357	44.31	5.80–882.69	413.75	77.401	0.000
	Multiple nodules	244	158.67	9.27–1210	480.64		
	Infiltrative	139	190.70	15.39–1097.22	514.60		
	Large tumor	252	993.00	79.95–3000.00	619.10		
TD^b^	Low	44	94.98	7.68–750.92	104.66	4.769	0.092
	Medium	111	86.59	4.33–780.26	92.46		
	High	17	63.94	3.34–64.84	70.67		
Child–Pugh^b^	A	585	77.02	6.56–1000.00	442.59	51.720	0.000
	B	289	686.4	25.07–2644.37	557.29		
	C	116	1000	99.57–1210	608.37		
BCLC^b^	0	22	12.65	4.34–70.97	233.55	54.373	0.000
	A	201	39.50	5.16–541.49	289.41		
	B	221	334.60	17.50–1955.01	394.89		
	C	257	586.10	28.19–1210	405.95		
	D	34	1000	158.68–3000.00	457.93		
Clinical staging^b^	I	53	9.97	3.64–193.50	99.79	22.725	0.000
	II	37	213.21	6.56–843.00	140.82		
	III	105	155.80	14.02–1210.00	146.92		
	IV	92	887.50	23.87–1210.00	167.41		

^a^Mann–Whitney U

^b^Kruskai–Wallis H

PT: Thrombosis of portal vein; Metastasis: lymph nodes or distant organ metastasis; TD: tumor differentiation; BCLC: Barcelona clinic liver cancer staging

### Association between serum AFP status and hepatitis B biomarkers in HCC patients

Patients with HBsAg and HBeAg positive and HBV DNA amount > 500 IU/mL had significantly higher serum AFP levels (p = 0.000 for all) (Table 3).

**Table 3:** Association between serum AFP levels and hepatitis B biomarkers in liver cancer patients (Mann–Whitney)

		N	Median	P25–P75	Mean rank	Z	*P*
HBsAg	Negative	177	20.23	3.32–1000.00	371.13	− 6.014	0.000
	Positive	793	239.95	15.55–1210.00	511.03		
HBeAg	Negative	849	159.21	7.93–1210.00	475.31	− 3.527	0.000
	Positive	125	417.20	41.91–3000.00	570.26		
HBV DNA (copies)	≤ 500	389	110.69	7.35–1072.50	349.76	− 4.456	0.000
(IU/ml)	> 500	380	501.23	29.28–1598.65	421.07		

### Association between serum AFP status and other tumor markers in HCC patients

**Fig. 1:**
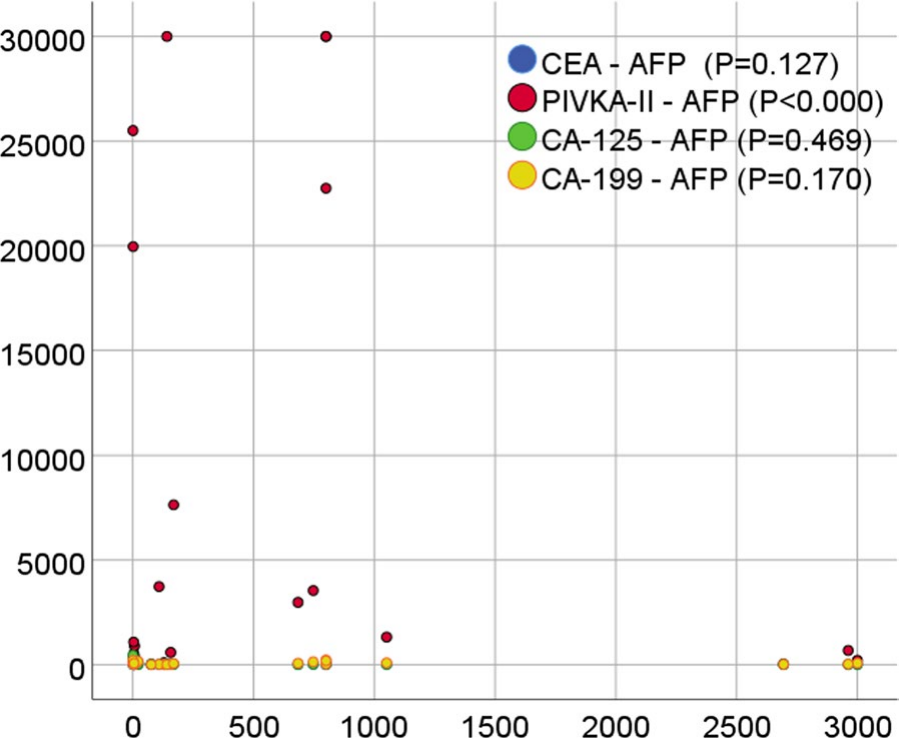
The overlay scatter of serum AFP and other tumor markers (PIVKA-II, CEA, CA-125, and CA-199). Figure shows the association between serum AFP levels and PIVKA-II, CEA, CA-125, and CA-199 in HCC patients using the overlay scatter plot, a p value is displayed

Patients with high serum AFP levels had higher serum PIVKA-II (p < 0.000). There is no significant correlation between serum AFP and CEA, CA-125, and CA-199 levels (p = 0.127, 0.469, and 0.170, respectively). Additional file 2: Table S2. The Overlay Scatter of serum AFP levels with CEA, PIVKA-II, CA-125, and CA-199 are shown in Fig. 1 for further demonstration.

### Association between serum AFP status and liver function biomarkers in HCC patients

Patients with high serum AFP levels had higher serum alanine aminotransferase (ALT), γ-glutamyl transpeptidase (γ-GT), aspartate aminotransferase (AST), alpha-L-fucosidase (AFU) activities (p = 0.000 for all), and higher γ-GT/ALT ratio (p = 0.000). Patients with high serum AFP levels also had higher serum direct bilirubin (DBIL) and indirect bilirubin (IBIL) levels (p = 0.000 and p = 0.015, respectively) and fibrinogen (p = 0.005) as well as D-Dimer (p = 0.000). There was no significant association between serum AFP levels and serum homocysteine (Hcy, p = 0.445), ferritin (SF, p = 0.830), total protein (TP, p = 0.093), and albumin (ALB, p = 0.496). The prothrombin time did not show significant different either (p = 0.204) between AFP negative and positive patients (Table 4).

**Table 4:** Association between serum AFP levels and liver function markers in patients with AFP negative and AFP positive (Mann–Whitney)

	AFP	n	Median	P25–P75	Mean rank	Z	*P*
Hcy (µg/L)	–	88	15.10	12.10–20.85	87.94	− 0.764	0.445
	+	93	15.60	12.40–21.80	93.89		
SF (µg/L)	–	142	560.79	235.18–1125.95	197.35	− 0.214	0.830
	+	142	575.8	268.20–1195.70	199.92		
ALT (U/L)	–	312	32.00	20.00–52.00	418.30	− 5.733	0.000
	+	677	42.00	27.00–68.00	530.35		
γ-GT (U/L)	–	312	71.00	38.00–148.50	388.43	− 7.965	0.000
	+	677	142.00	67.00–269.00	544.11		
γ-GT/ALT	–	312	2.24	1.32–4.82	442.96	− 3.889	0.000
	+	677	3.25	1.67–5.80	518.98		
AST (U/L)	–	313	41.00	27.00–63.00	378.04	− 8.815	0.000
	+	678	64.50	39.00–125.25	550.45		
AFU (U/L)	–	297	27.40	22.80–34.00	353.55	− 9.265	0.000
	+	654	36.33	27.18–50.03	531.61		
DBIL (μmol/L)	–	311	4.90	3.30–9.70	437.47	− 4.258	0.000
	+	677	6.50	4.00–13.90	520.7		
IBIL (μmol/L)	–	311	11.40	7.50–17.30	461.86	− 2.437	0.015
	+	677	12.30	8.20–20.65	509.49		
TP (g/L)	–	312	67.10	62.23–71.98	471.6	− 1.678	0.093
	+	675	67.40	62.80–73.00	504.35		
ALB (g/L)	–	312	35.85	30.80–40.68	504.17	− 0.686	0.493
	+	677	35.10	30.80–39.90	490.77		
PT (sec)	–	311	14.20	13.40–15.40	475.56	− 1.271	0.204
	+	673	14.40	13.60–15.40	500.33		
INR	–	234	1.13	1.05–1.23	387.1	− 0.475	0.635
	+	551	1.14	1.06–1.24	395.5		
Fibrinogen		233	3.09	2.51–4.30	358.29	− 2.786	0.005
		552	3.48	2.67–4.54	407.65		
D-dimer		216	0.89	0.45–2.53	326.11	− 3.592	0.000
		523	1.35	0.68–2.94	388.13		

AFP (−): < 20 µg/L; AFP (+): ≥ 20 µg/L; Hcy: homocysteine; SF: serum ferritin; ALT: alanine aminotransferase; γ-GT: γ-glutamyl transpeptidase; AST: Aspartate aminotransferase; AFU: alpha-L-fucosidase; DBIL: direct bilirubin; IBIL: indirect bilirubin; TP: total protein; ALB: albumin; PT: prothrombin time; INR: international normalized ratio

### Association between serum AFP status and hematology parameters in HCC patients

Table 5 shows that serum AFP status is positively associated with white blood cell (WBC) count, neutrophils, monocytes, and platelets (p = 0.002, 0.000, 0.020, and 0.006, respectively). Serum AFP positive patients have lower lymphocyte percentage than negative patients (p = 0.000), thus, the neutrophils to lymphocytes ration (NLR) is higher in serum AFP positive patients (p = 0.000).

**Table 5:** Association between serum AFP levels and hematology parameters (Mann–Whitney)

	AFP	N	Median	P25–P75	Mean rank	Z	*P*
WBC	–	313	5.91	4.45–7.68	487.43	− 3.068	0.002
	+	679	6.45	5.00–8.38	542.28		
Neu	–	313	3.48	2.45–5.46	444.02	− 3.917	0.000
	+	679	4.17	3.00–5.99	520.69		
Neu%	–	313	63.00	55.00–73.00	495.6	− 3.933	0.000
	+	679	67.00	59.00–74.10	538.2		
Lym	–	313	1.43	1.00–1.89	508.97	− 0.931	0.352
	+	679	1.37	1.00–1.86	490.75		
Lym%	–	313	26.00	16.00–33.15	547.13	− 3.779	0.000
	+	679	22.00	15.00–29.4	473.16		
NLR	–	313	2.36	1.67–4.52	444.65	− 3.870	0.000
	+	679	3.04	2.03–4.89	520.4		
Mon	–	313	0.46	0.32–0.64	465.43	− 2.319	0.020
	+	679	0.5	0.37–0.69	510.82		
Mon%	–	313	8.00	6.00–10.00	493.93	− 0.192	0.848
	+	679	8.00	6.20–10.00	497.68		
PLT	–	236	166	125–226	361.68	− 2.755	0.006
	+	555	191	127–260	410.6		

AFP (−): < 20 µg/L; AFP (+): ≥ 20 µg/L; WBC: white blood cell count; Neu: neutrophil count; Neu%: percentage of neutrophils; Lym: lymphocyte count; Lym%: percentage of lymphocytes; NLR: neutrophil to lymphocyte ratio; Mon: monocyte count; Mon%: percentage of monocytes; PLT: platelet count

## Discussion

AFP is a major plasma protein produced by the yolk sac and the liver during fetal life. AFP expression in adults is often associated with hepatoma or teratoma [26].

The underlying mechanism of AFP expression status in HCC and its role in the development and prognosis is still unclear; promoter polymorphism or silencing of *AFP* gene is believed to be the major reason [27, 28]. Understanding the clinical pathologic characteristics of liver cancer patients with low and high serum AFP could be helpful for clinical management and prognostication of this deadly disease.

### Serum AFP status among patient groups

In this observation, we found that serum AFP levels in HCC patients were significantly higher than in chronic hepatitis B, decompensated hepatic cirrhosis, and hepatitis B virus asymptomatic carrier patients. This further indicates that tumor cells secret AFP into the serum, the detailed mechanism is still unclear. Cellular membrane damage and/or eruption of tumor cells are believed to involved in the pathogenesis [29]. One study mentioned that HCC patients with negative AFP tended to be older males with less HBV infection, more non-viral etiology, and less cirrhosis [30]. Table 1 and Additional file 1: Figure S2 A indicate a trend from high to low in median AFP levels of different patient groups, consistent with this report.

### High serum AFP level was associated with unfavorable prognostic factors

We also found that patients with larger tumors, hepatic cirrhosis, portal vein thrombosis, metastasis, and massive hepatocellular carcinomas had higher serum AFP levels. These suggest that patients with higher serum AFP levels tend to have poor clinical outcome [31–34]. It is also possible that higher burden of disease exists in these patients accounting for the increased AFP levels.

### Association of serum AFP levels between Child–Pugh score, BCLC stage, and clinical stage

Serum AFP levels were significantly higher in patients with high Child–Pugh score, high BCLC stage, and advanced clinical stage. Lim et al. reported that AFP levels and Child–Pugh scores were associated with brain metastasis in HCC patients [35]. Liu et al. reported that based on BCLC staging, serum AFP response was correlated with the efficacy of transarterial chemoembolization in HCC patients [36]. Incorporation of serum AFP into the BCLC staging system was helpful in predicting the outcome of HCC patients, and AFP positive was an independent poor prognostic factor [37]. Gomaa et al. found that the addition of AFP and ascites to the BCLC staging system improved the prognostic prediction for early and intermediate stages of liver cancer patients [38].

### Patients with HBsAg and HBeAg positive, and high HBV DNA levels had significantly higher serum AFP levels

Again, our observation further confirmed that serum AFP levels were associated with hepatitis B viral biomarkers positivity as reported previously [39]. HCC patients with positive HBsAg and HBeAg, high levels of HBV DNA had higher serum AFP levels. This further indicates that active viral replication and chronic liver disorder and hepatocyte damage is occurring in liver cancer patients [40, 41].

### Association between serum AFP levels and other tumor markers

Patients with high serum AFP levels also had higher serum PIVKA-II. PIVKA-II is usually used in addition to AFP measurement for HCC diagnosis [42]. However, in this study, we found that there was a positive association between serum AFP and PIVKA-II levels in HCC patients. Si et al. reported that combined analyses of serum AFP and PIVKA-II increased the diagnostic performance for HBV-related HCC [43]. We found that there was no association between serum AFP and CEA, CA-125, and CA-199 levels. Report mentioned that elevated serum AFP and CEA were found in advanced hepatocellular carcinoma with extrahepatic metastasis [44]. Gou et al. found that both serum CEA and CA-199 increased but not AFP in primary adenosquamous carcinoma of the liver [45]. Serum CEA level may differ according to HCC subgroups which needs to be further investigated [46].

### Association between serum AFP status and liver function, hemostatic status in HCC patients

As described above, serum AFP status was associated with Child–Pugh score. We further analyzed individual elements of the Child–Pugh scoring system, the ALT, AST, AFU, γ-GT, γ-GT/ALT ratio, DBIL, IBIL levels, fibrinogen, and D-dimer levels with serum AFP status. Results showed that patients with high serum AFP levels had higher serum ALT, AST, AFU, γ-GT, γ-GT/ALT ratio, DBIL, and IBIL levels, as well as fibrinogen and D-dimer levels. These results further confirmed the clinical usage of Child–Pugh scoring system for liver cancers. Elevated fibrinogen and D-dimer levels may suggest a potential deep venous thrombosis, or other complications in advanced tumors including liver cancer.

### Association of serum AFP status with hematology parameters

Our observation found that serum AFP status was positively associated with peripheral blood WBC, neutrophils, monocytes, platelets, and the neutrophils to lymphocytes ratio (NLR). These parameters may suggest that an inflammatory or infection condition occurred in patients with high serum AFP levels, since the AFP status was associated with unfavorable clinical pathologic features as described above. Shiraki et al. reported that elevated NLR is predictive of a poor survival in patients with primary HCC showing normal AFP levels [47]. Another report on a systemic review of the literature showed that NLR and albumin in HCC predicted survival better than the conventional AFP [48]. Our finding on NLR was opposite to that of Huang et al. [18].

## Conclusions

In summary, the results especially the laboratory parameters observed in our study in AFP negative HCC patients could be used for diagnosis and prognostic evaluation of these patients. HBV DNA viral load and hematologic indices are especially new parameters in associating with serum AFP status in HCC.

## Supplementary Information

**Additional file 1.** Patients’ demographics and the association between serum AFP levels and other tumor markers in patients with AFP negative and AFP positive.

**Additional file 2.** Distribution of AFP in patient groups and the comparison of serum AFP levels among patient groups and among clinical pathologic features.

## Data Availability

Data is available by contacting Huayi Huang at Henry.Huang@Mindray.com.

## Abbreviations

HCC: Hepatocellular carcinoma
PLC: Primary liver cancer
CCA: Cholangiocarcinoma
H-ChC: Hepatocellular-cholangiocarcinoma
DHC: Decompensated hepatic cirrhosis
CHB: Chronic hepatitis B
AsC: Hepatitis B virus asymptomatic carrier
PVT: Portal vein thrombosis
MHC: Massive hepatocellular carcinoma
BCLC: Barcelona clinic liver cancer staging
CEA: Carcinoembryonic antigen
PIVKA-II: Protein induced by vitamin K absence or antagonist-II
CA-125: Cancer antigen 125
CA-199: Cancer antigen 199
Hcy: Homocysteine
SF: Serum ferritin
ALT: Alanine aminotransferase
γ-GT: γ-Glutamyl transpeptidase
AST: Aspartate aminotransferase
AFU: Alpha-L-fucosidase
DBIL: Direct bilirubin
IBIL: Indirect bilirubin
TP: Total protein
ALB: Albumin
PT: Prothrombin time
INR: International normalized ratio
WBC: White blood cell count
Neu: Neutrophil count
Neu%: Percentage of neutrophils
Lym: Lymphocyte count
Lym%: Percentage of lymphocytes
NLR: Neutrophil to lymphocyte ratio
Mon: Monocyte count
Mon%: Percentage of monocytes
