# Supplementary material for: A comparison of clinical pathologic characteristics between alpha-fetoprotein negative and positive hepatocellular carcinoma patients from Eastern and Southern China
Source: BMC Gastroenterol. 2022 Apr 23;22:202. doi: 10.1186/s12876-022-02279-w (PMC9034573; doi:10.1186/s12876-022-02279-w)
Supplement: Supplementary file 1 — Additional file 1. Patients’ demographics and the association between serum AFP levels and other tumor markers in patients with AFP negative and AFP positive. [file 12876_2022_2279_MOESM1_ESM.pdf]

Figure S1 Distribution of AFP in patient groups

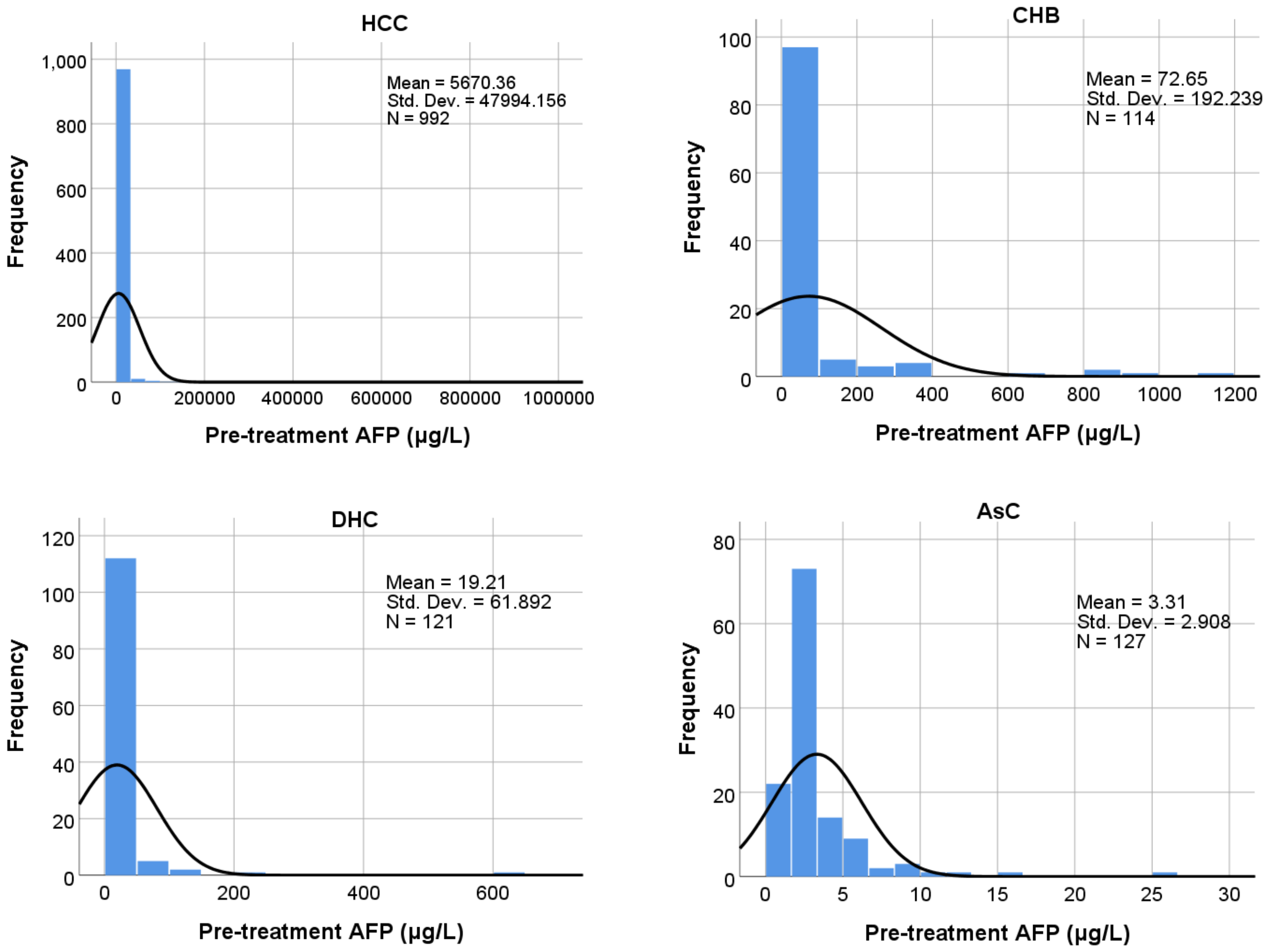

Figure S1 Distribution of serum AFP in patient groups

Figure S1 shows that the distribution of AFP in all patient groups is in the lower level range (left side on the histogram), thus leading to a skewed normality test result.

Figure S2 Comparison of serum AFP levels among patient groups, clinical pathologic features

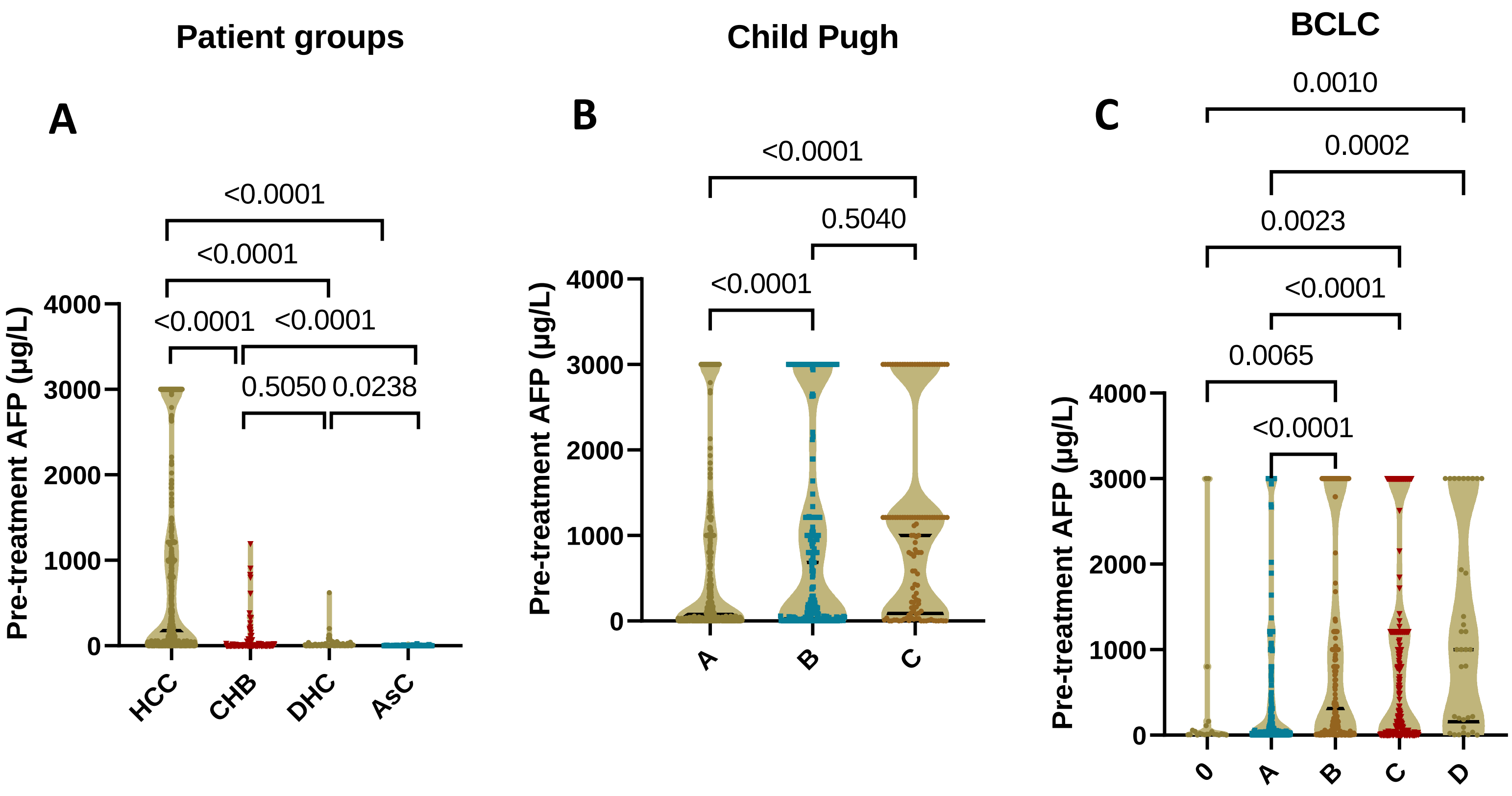

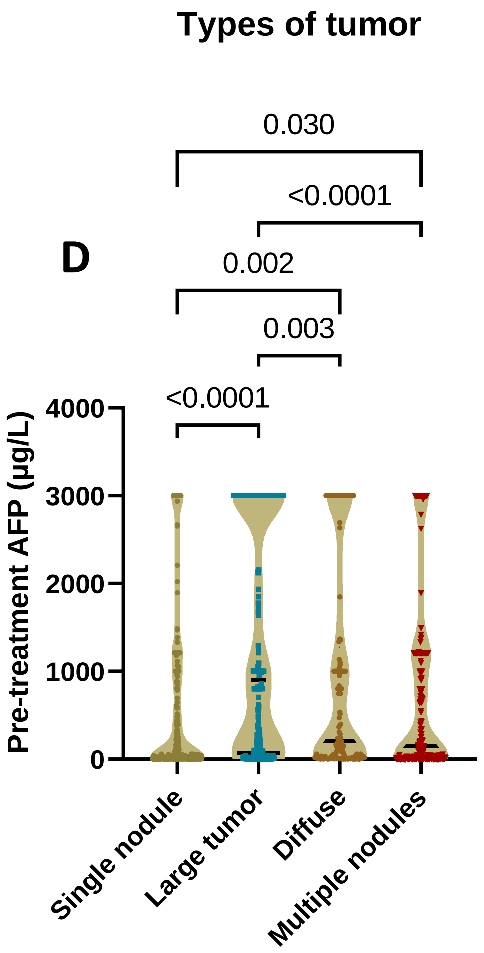
Types of tumor
D
0.030
<0.0001
0.002
0.003
<0.0001
Pre-treatment AFP (μg/L)
4000
3000
2000
1000
0
Single nodule
Large tumor
Diffuse
Multiple nodules

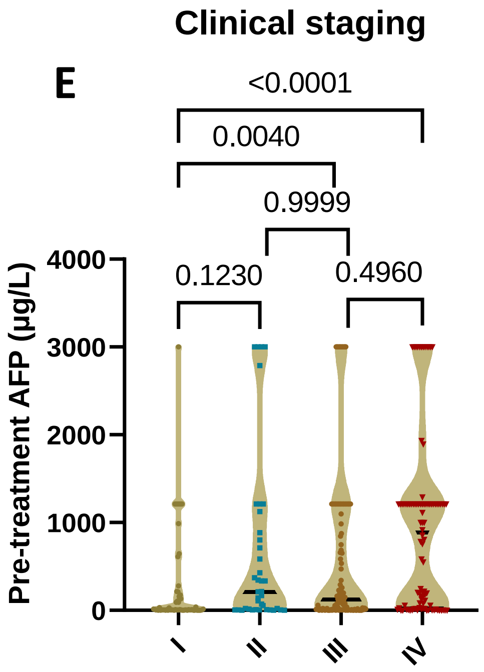
Clinical staging
E
<0.0001
0.0040
0.9999
0.1230
0.4960
Pre-treatment AFP (μg/L)
4000
3000
2000
1000
0
I
II
III
IV

Figure S2 Comparison of serum AFP levels among patient groups, clinical pathologic features

Figure S2 further demonstrate the comparison results of serum AFP levels among patient groups, among different clinical patho logic features. A p value is marked for those parameters with significant difference.
