# Supplementary material for: A comparison of clinical pathologic characteristics between alpha-fetoprotein negative and positive hepatocellular carcinoma patients from Eastern and Southern China
Source: BMC Gastroenterol. 2022 Apr 23;22:202. doi: 10.1186/s12876-022-02279-w (PMC9034573; doi:10.1186/s12876-022-02279-w)
Supplement: Supplementary file 2 — Additional file 2. Distribution of AFP in patient groups and the comparison of serum AFP levels among patient groups and among clinical pathologic features. [file 12876_2022_2279_MOESM2_ESM.pdf]

Table S1 Patients' demographics

		n	Median of age	Age rang
Hepatoma				
HCC	M	861	55	23-89
	F	131	60	20-83
	Total	992		
DHC	M	85	53	30-86
	F	36	59	40-85
	Total	121		
CHB	M	80	36.5	14-72
	F	34	44.5	21-68
	Total	114		
AsC	M	68	39.5	5-67
	F	59	38	25-80
	Total	127		

HCC: Hepatocellular carcinoma; DHC: Decompensated hepatic cirrhosis; CHB: Chronic hepatitis B; AsC: Hepatitis B virus asymptomatic carrier.

Table S2 Association between serum AFP levels and other tumor markers in patients with AFP negative and AFP positive (Mann-Whitney)

	AFP	n	Median	P25-P75	Mean Rank	Z	*P*
CEA (μg/L)	-	267	2.26	1.2-3.5	429.18	-1.525	0.127
	+	554	2.12	1.23-3.19	402.24		
PIVKA II (mAU/mL)	-	41	124	37-436	41.26	-4.926	0.000
	+	88	2387	175-14574	76.06		
CA-125 (μg/L)	-	124	33.01	10.70-182.35	174.06	-0.724	0.469
	+	234	32.05	12.70-276.06	182.38		
CA-199 (μg/L)	-	188	20.38	9.37-40.75	277.17	-1.374	0.170
	+	393	21.65	11.94-48.42	297.62		

AFP (-): AFP <20 μg/L； AFP (+): AFP ≥20 μg/L; CEA: carcinoembryonic antigen; PIVKA-II: protein induced by vitamin K absence or antagonist-II; CA-125: cancer antigen 125; CA-199: cancer antigen 199.
